# Transplantation of Wnt5a-modified NSCs promotes tissue repair and locomotor functional recovery after spinal cord injury

**DOI:** 10.1038/s12276-020-00536-0

**Published:** 2020-12-14

**Authors:** Xiang Li, Zhiming Peng, Lingli Long, Xiaofang Lu, Kai Zhu, Ying Tuo, Ningning Chen, Xiaoyang Zhao, Le Wang, Yong Wan

**Affiliations:** 1 Department of Spine Surgery, The First Affiliated Hospital, Sun Yat-Sen University, 510080 Guangzhou, Guangdong China; 2 Translational Medicine Center, The First Affiliated Hospital, Sun Yat-Sen University, 510080 Guangzhou, Guangdong China; 3 Department of Pathology, The Seventh Affiliated Hospital, Sun Yat-Sen University, 518107 Shenzhen, Guangdong China; 4 Department of Pathology, The First Affiliated Hospital, Sun Yat-Sen University, 510080 Guangzhou, Guangdong China; 5 Department of Spine Surgery, The Seventh Affiliated Hospital, Sun Yat-Sen University, 518000 Shenzhen, Guangdong China; 6 Guangdong Province Key Laboratory of Orthopaedics, 510080 Guangzhou, Guangdong China

**Keywords:** Neural stem cells, Neuroscience

## Abstract

Traditional therapeutic strategies for spinal cord injury (SCI) are insufficient to repair locomotor function because of the failure of axonal reconnection and neuronal regeneration in the injured central nervous system (CNS). Neural stem cell (NSC) transplantation has been considered a potential strategy and is generally feasible for repairing the neural circuit after SCI; however, the most formidable problem is that the neuronal differentiation rate of NSCs is quite limited. Therefore, it is essential to induce the neuronal differentiation of NSCs and improve the differentiation rate of NSCs in spinal cord repair. Our results demonstrate that both Wnt5a and miRNA200b-3p could promote NSC differentiation into neurons and that Wnt5a upregulated miRNA200b-3p expression through MAPK/JNK signaling to promote NSC differentiation into neurons. Wnt5a could reduce RhoA expression by upregulating miRNA200b-3p expression to inhibit activation of the RhoA/Rock signaling pathway, which has been reported to suppress neuronal differentiation. Overexpression of RhoA abolished the neurogenic capacity of Wnt5a and miRNA200b-3p. In vivo, miRNA200b-3p was critical for Wnt5a-induced NSC differentiation into neurons to promote motor functional and histological recovery after SCI by suppressing RhoA/Rock signaling. These findings provide more insight into SCI and help with the identification of novel treatment strategies.

## Spinal cord injury: Boost for neural stem cell therapies

Incorporating key molecules into neural stem cells enhances their ability to differentiate correctly and promote repair following spinal cord injury. Spinal cord injuries can have a debilitating effect on patients’ lives, yet there are no therapies that fully restore movement and sensation. Therapies based on neural stem cells (NSCs) show promise, but initial studies show many NSCs differentiate into astrocytes, supportive cells that do not conduct nerve impulses, instead of neurons, leading to treatment failure. Yong Wan and Le Wang at Sun Yat-sen University in Guangzhou, China, and co-workers demonstrated that adding a protein called Wnt5a and a specific microRNA molecule to NSCs significantly increases differentiation into neurons. Wnt5a suppresses a signalling pathway that otherwise interferes with NSC differentiation. Experiments on rat models showed that the therapy improved locomotor function and tissue repair after injury.

## Introduction

Spinal cord injury (SCI) is considered to be a refractory disease with devastating physical, psychosocial, and vocational implications for patients and caregivers despite enormous advances in medical and surgical treatments^1,2^. The incidence of SCI around the world is on average 40–80 per million, with 250,000 to 500,000 injuries occurring each year globally and imposing a large financial burden^1,3^. Therapeutic interventions include surgical decompression, therapeutic hypothermia, and pharmacotherapy aimed at reducing tissue damage and improving patient quality of life^4,5^. However, none of these therapeutic strategies are sufficient to repair the interruption of the neuroanatomical circuit. Consequently, the failure of axonal connections and neural regeneration in the injured central nervous system (CNS) remains a challenge for researchers^1^.

Cell transplantation has been considered a potential strategy to repair the neural circuit after SCI^6,7^. Several studies have reported that neural stem cell (NSC) transplantation results in partial repair due to the neuronal differentiation of NSCs^8,9^. However, in most SCI cases, only a few exogenous NSCs differentiate into neurons, while most of them differentiate into astrocytes, which is a disadvantage in spinal cord repair^10,11^. Therefore, the challenging points are increasing the differentiation rate of neurons and promoting the differentiated NSCs to reconnect the neural circuit.

One of most important mechanisms of cell transplantation therapy is promoting neuronal-oriented differentiation to rebuild the neural circuit^1^. Wnt signaling is critical in modulating many biological processes, including neuronal differentiation and regeneration. Many studies of SCI have demonstrated that canonical Wnt/β-catenin signaling has a neuroprotective effect and induces axonal and neural regeneration effectively^12–15^; however, Wnt/β-catenin has been reported to induce tumorigenesis under specific conditions, and the neural effect of β-catenin is suppressed by other pathways, such as Notch signaling^16–20^. Thus, the clinical application of Wnt/β-catenin signaling is greatly limited. Recently, noncanonical Wnt proteins, such as Wnt4, Wnt5a, and Wnt11, have gained growing attention as attractive factors for neuronal differentiation. Noncanonical Wnt signaling, including the Wnt/Ryk, Wnt/Ca^+^, and Wnt/JNK pathways, has been reported to have a positive effect on neuronal differentiation^21–23^, but the therapeutic effect and mechanism in promoting neuronal differentiation are still unclear.

MicroRNAs (miRNAs) are small noncoding RNA molecules that have been shown to play critical roles in regulating gene expression at the posttranscriptional level in many cellular processes, including neural development, tumor metastasis, cell proliferation, apoptosis, and differentiation^24^. Previous studies have reported that RhoA is the target gene of miRNA200b-3p (mi200b-3p) and that RhoA expression is downregulated by mi200b-3p^25^. In addition, the RhoA/Rock1 pathway has been recognized as a negative neurogenesis pathway that has adverse effects on neuronal differentiation and tissue repair in SCI^26^. Thus, confirming the mechanism of action of mi200b-3p/RhoA in neuronal differentiation is necessary but has yet to be achieved.

The purpose of the current study was to investigate the neurogenic capacity of Wnt5a and the potential of Wnt5a for application in NSC transplantation in SCI and explore the underlying mechanism, which may provide useful information for translational applications.

## Methods and materials

A detailed description of all materials and methods used is provided in the Supplementary Materials and Methods, as follows: NSC isolation, culture, and transfection, lentiviral vector construction, transduction, transfection of mi200b-3p mimics and inhibitors, pharmaceutical inhibition, real-time quantitative reverse transcription PCR (RT-qPCR), western blot analysis, luciferase reporter assay, immunofluorescence, surgical procedures and cell transplantation, functional assessment, histological analyses and statistical analyses.

### NSC isolation and culture

NSCs were obtained from the fetal brain of embryonic day 14 rats, which were extracted from pregnant Sprague-Dawley (SD) rats (Laboratory Animal Center of Sun Yat-Sen University, Guangzhou, China)^27–29^. NSCs were plated in a T25 culture flask (Corning, Acton, MA, 430639) containing maintenance medium consisting of Dulbecco’s modified Eagle’s medium/F12 nutrient mixture, 2% B27, 1% penicillin/streptomycin, 1% l-glutamine (Invitrogen, Carlsbad, CA, 11320033), 20 ng/mL fibroblast growth factor-2 (FGF-2) and 20 ng/mL epidermal growth factor (EGF) (PeproTech, Rocky Hill, NJ, 96–400–29, 96-AF-100–15). NSCs were cultured at 37 °C in 5% CO_2_ and were passaged via weekly digestion with Accutase (Millipore, Bedford, MA, SCR005) in the medium described above. All NSCs used in this study were between passages 2 and 4.

To induce neuronal differentiation, cells were plated at a density of 2 × 10^5^ cells/well in 6- or 12-well tissue culture plates and allowed to adhere for 24 h at 37 °C, at which time cells were switched to neuronal differentiation medium consisting of basic medium supplemented with 2% B27, 1% penicillin/streptomycin, and 1% l-glutamine. The medium was changed every 2–3 days^29^.

### Animal experiments

Adult female SD rats (weighing 200–220 g, supplied by the Experimental Animal Center of Sun Yat-Sen University, Guangzhou, China) were used to establish an SCI animal model^27–29^. Behavioral and histological analyses were performed using previously described standard methods^30–33^ and detailed in online supplementary materials. All procedures performed on experimental animals were approved by the Animal Care and Use Committee of Sun Yat-Sen University and were conducted in accordance with the Guide to the Care and Use of Experimental Animals by the National Research Council (1996, United States).

## Results

### miRNA200b-3p is critical for Wnt5a promoting neuronal differentiation

We first determined the neuronal differentiation capacity of Wnt5a and mi200b-3p in NSCs. NSCs were treated with Wnt5a (10 ng/mL) or transfected with the mi200b-3p mimic. The immunofluorescence results showed that the number of β3-tubulin- and MAP2-positive cells was significantly increased by Wnt5a and mi200b-3p, while the number of GFAP-positive cells was not increased (Supplementary Figs. S1a, b and S2a, b). Similar results of RT-qPCR and WB revealed the expression of neurogenic markers, including β3-tubulin, MAP2 and GFAP, at the mRNA and protein levels (Supplementary Figs. S1c, d and S2c, d). These results suggest that Wnt5a and mi200b-3p were able to promote neuronal differentiation.

**Fig. 1:**
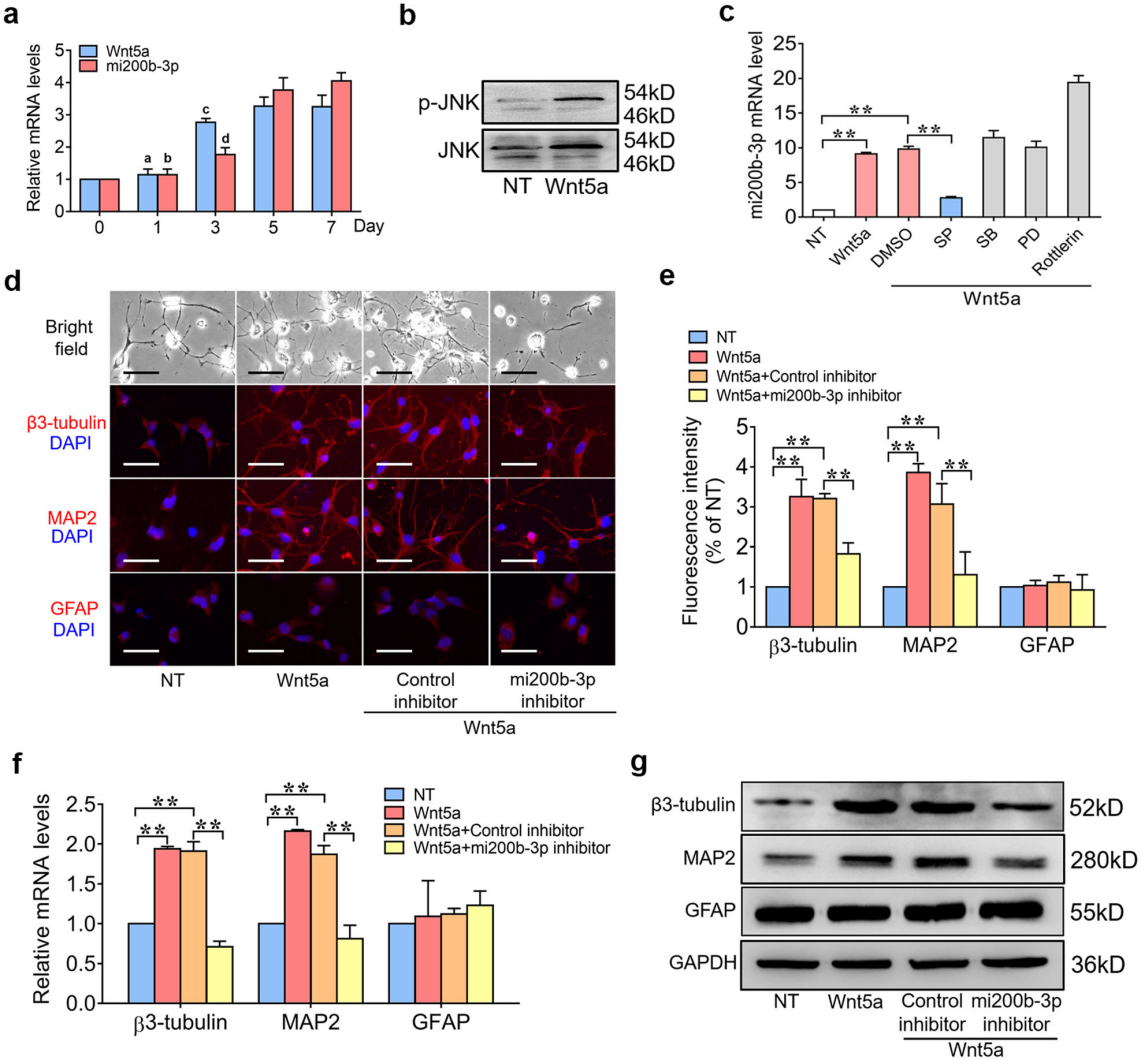
miRNA200b-3p is critical for Wnt5a to promote neuronal differentiation. **a** RT-qPCR analyses of Wnt5a and mi200b-3p expression with neuronal differentiation over time. **b** Western blot analyses of p-JNK expression in NSCs treated with Wnt5a. **c** RT-qPCR analyses of mi200b-3p in NSCs treated with different pathway inhibitors and then stimulated with Wnt5a. **d** and **e** Immunofluorescence staining of NSCs transfected with an mi200b-3p inhibitor and then stimulated with Wnt5a. **f** and **g** RT-qPCR and western blot analyses of neurogenic marker expression in NSCs transfected with an mi200b-3p inhibitor and then stimulated with Wnt5a. SP: JNK inhibitor; PD: ERK inhibitor; SB: p38 inhibitor; Rottlerin: PKCδ inhibitor (the data are presented as the mean ± SD from one representative experiment of three independent experiments performed in triplicate. ^**a, b**^*P* < 0.01 compared between 3 days, 5 days, and 7 days; ^**c, d**^*P* < 0.05 compared between 5 days and 7 days; **P < 0.01 compared between groups.).

We next determined whether mi200b-3p was involved in Wnt5a-induced neurogenesis. We first examined the expression of Wnt5a and mi200b-3p in the process of neuronal differentiation. The RT-qPCR results showed that both Wnt5a and mi200b-3p expression significantly increased with the duration of neuronal differentiation (Fig. 1a). The WB results confirmed that Wnt5a could promote the activation of MAPK/JNK pathways (Fig. 1b). Furthermore, NSCs were treated with pharmaceutical inhibitors of MAPKs and PKCδ and then stimulated with Wnt5a for 24 h. mi200b-3p expression was then detected with RT-qPCR, and the results showed that inhibiting the JNK pathway significantly decreased mi200b-3p expression at the mRNA level (Fig. 1c), suggesting that Wnt5a upregulated mi200b-3p expression through the MAPK/JNK pathway. To further determine whether mi200b-3p was involved in Wnt5a-induced neurogenesis, a target-specific inhibitor was transfected into NSCs to suppress mi200b-3p expression, and then the cells were treated with Wnt5a. The immunofluorescence results showed that the number of β3-tubulin- and MAP2-positive cells was significantly decreased by the mi200b-3p inhibitor, while the number of GFAP-positive cells was not increased (Fig. 1d, e). Similar results of RT-qPCR and WB showed the expression of neurogenic markers, including β3-tubulin, MAP2 and GFAP, at the mRNA and protein levels (Fig. 1f, g). These results suggest that mi200b-3p was critical to Wnt5a-induced neuronal differentiation.

### Wnt5a suppresses RhoA/Rock signaling by upregulating mi200b-3p

**Fig. 2:**
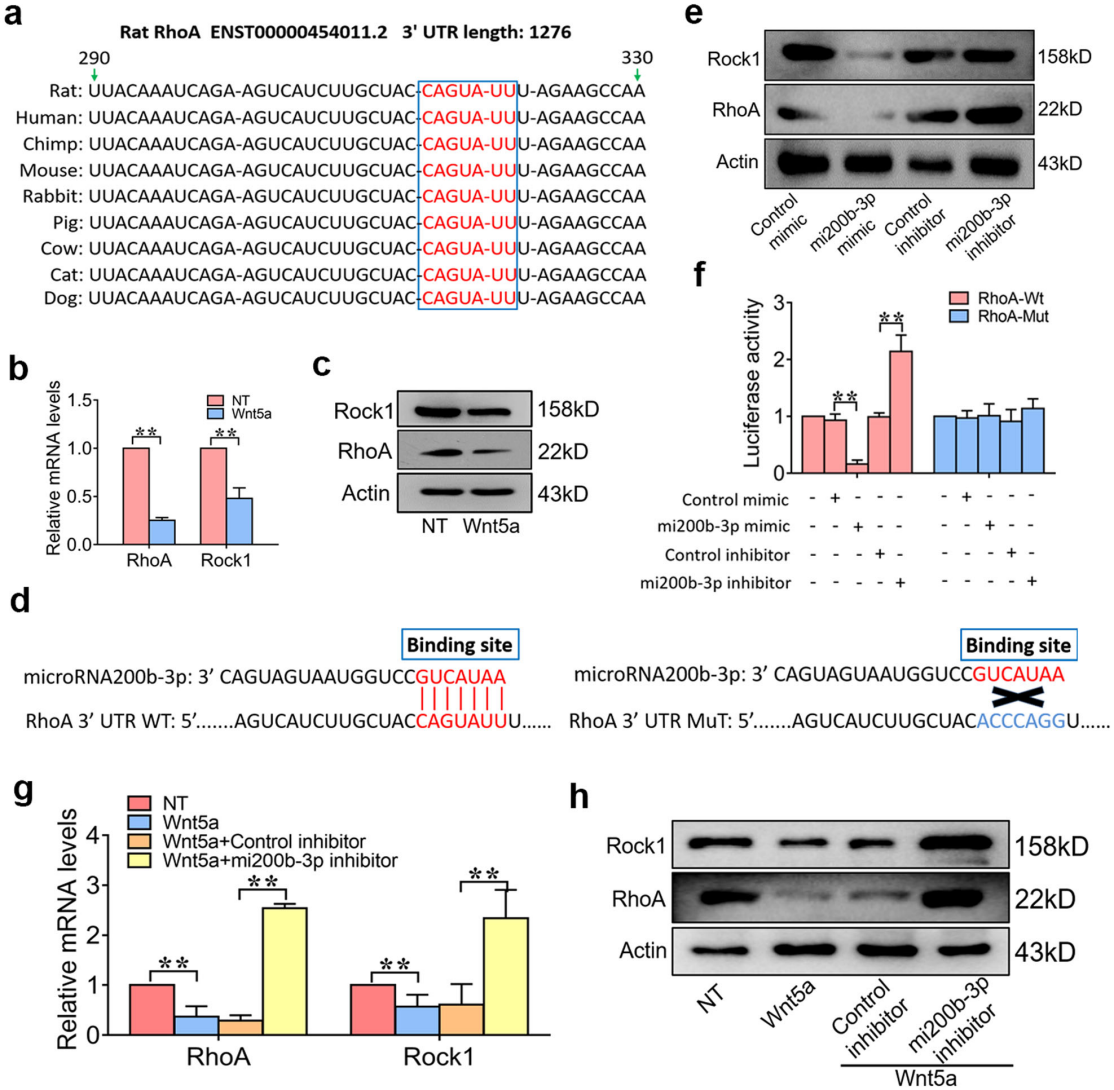
Wnt5a suppresses RhoA/Rock signaling by upregulating mi200b-3p. **a** A schematic diagram illustrating that the RhoA gene is widely conserved in many species. **b** and **c** RT-qPCR and western blot analyses of RhoA and Rock1 expression in NSCs stimulated with Wnt5a. **d** A schematic diagram illustrating the binding site of mi200b-3p in the RhoA sequence. **e** Western blot analyses of RhoA and Rock1 expression in NSCs treated with an mi200b-3p mimic and inhibitor. **f** mi200b-3p binding site-directed mutagenesis analysis of the RhoA promoter. **g** and **h** RT-qPCR and western blot analyses of RhoA and Rock1 expression in NSCs transfected with an mi200b-3p inhibitor and then stimulated with Wnt5a (the data are presented as the mean ± SD from one representative experiment of three independent experiments performed in triplicate. **P < 0.01 compared between groups.).

RhoA is a highly conserved gene in many species and modulates many biological processes, including intercellular adhesion, cell polarity, neural maintenance and differentiation, as well as gene expression^34^ (Fig. 2a). Activation of RhoA/Rock1 signaling in neural stem cells inhibits their survival and differentiation^26^. To investigate whether the neurogenic effect of Wnt5a depends on inhibition of the RhoA/Rock1 pathway, NSCs were treated with Wnt5a (10 ng/mL) for 3 days, and the RT-qPCR and WB results showed that Wnt5a suppressed RhoA and Rock1 gene expression (Fig. 2b, c). Moreover, we confirmed that RhoA is the target gene of mi200b-3p and that the binding site is from 290–329 bp (Fig. 2d). The western blot and luciferase activity results showed that the mi200b-3p mimic suppressed RhoA and Rock1 gene expression, while the mi200b-3p inhibitor increased RhoA and Rock1 expression at the protein level and transcriptional activity in NSCs (Fig. 2e, f). To further determine whether mi200b-3p is required for the inhibitory effect of Wnt5a on RhoA and Rock1 expression, an mi200b-3p inhibitor was used to suppress mi200b-3p expression in NSCs before Wnt5a stimulation. The results showed that with successful suppression of mi200b-3p, the inhibitory effect of Wnt5a on the expression of RhoA and Rock1 was significantly reduced (Fig. 2g, h). These results suggest that Wnt5a suppressed RhoA/Rock1 expression in an mi200b-3p-dependent manner.

### Overexpression of RhoA sabotaged the neuroinductive effects of Wnt5a/mi200b-3p

**Fig. 3:**
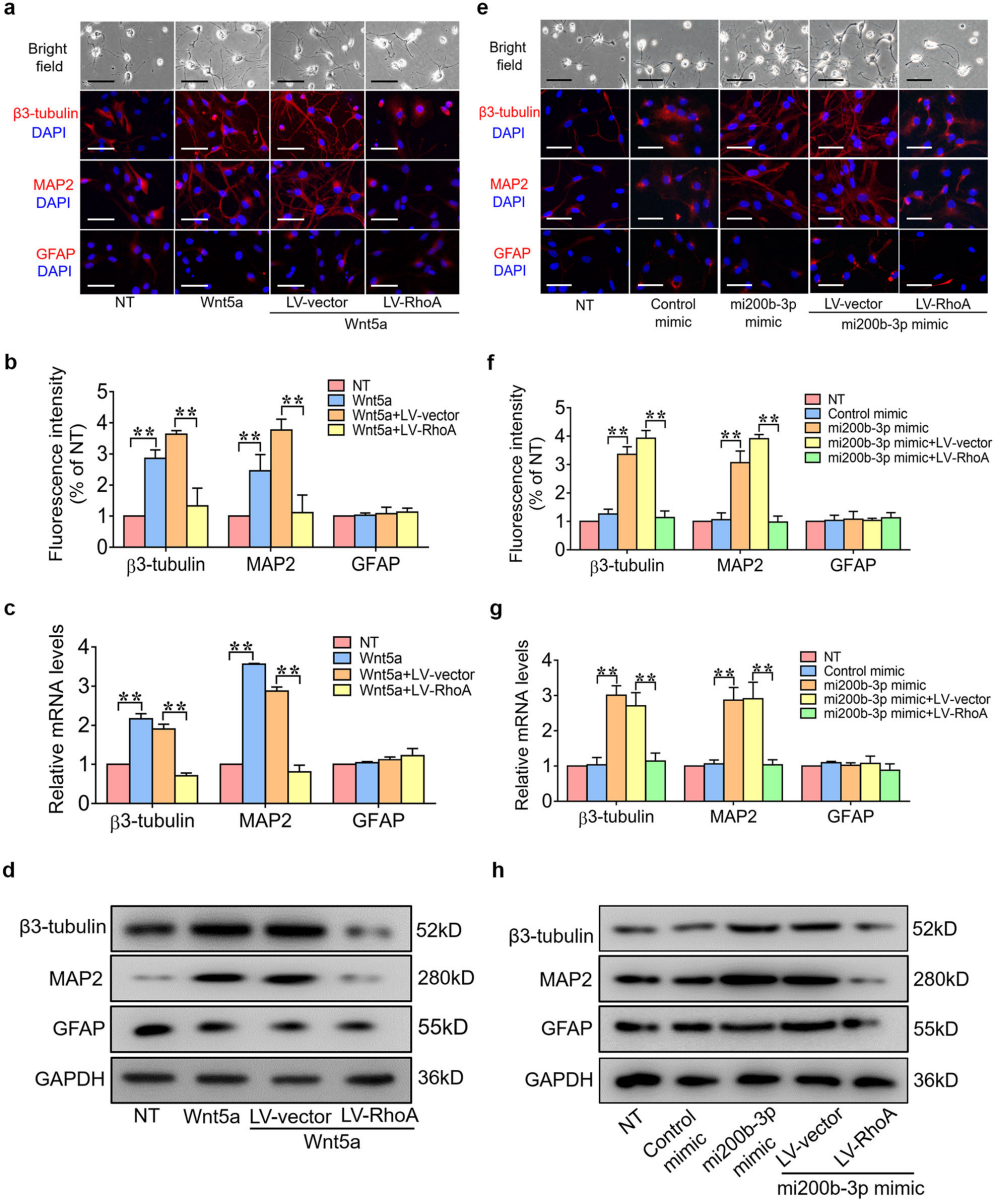
Overexpression of RhoA suppresses the neuroinductive effects of Wnt5a/mi200b-3p. **a** and **b** Immunofluorescence staining of NSCs transfected with LV-RhoA and then treated with Wnt5a. **c** and **d** RT-qPCR and western blot analyses of neurogenic marker expression in NSCs transfected with LV-RhoA and then treated with Wnt5a. **e** and **f** Immunofluorescence staining of NSCs transfected with the RhoA gene plasmid and treated with an mi200b-3p mimic. **g** and **h** RT-qPCR and western blot analyses of neurogenic marker expression in NSCs transfected with LV-RhoA and then treated with an mi200b-3p mimic. (The data are presented as the mean ± SD from one representative experiment of three independent experiments performed in triplicate. **P < 0.01 compared between groups.).

To further confirm that Wnt5a and mi200b-3p promote neuronal differentiation by suppressing RhoA expression, NSCs were transfected with RhoA gene lentivirus and then stimulated with a Wnt5a or mi200b-3p mimic. The immunofluorescence results showed that the number of β3-tubulin- and MAP2-positive cells was significantly increased by the Wnt5a and mi200b-3p mimics and significantly decreased by RhoA gene overexpression (Fig. 3a, b, e, f). Similar results of PCR and WB showed the expression of neurogenic markers, including β3-tubulin, MAP2 and GFAP, at the mRNA and protein levels (Fig. 3c, d, g, h).

### Wnt5a induces NSC differentiation into neurons to promote spinal cord repair in SCI

**Fig. 4:**
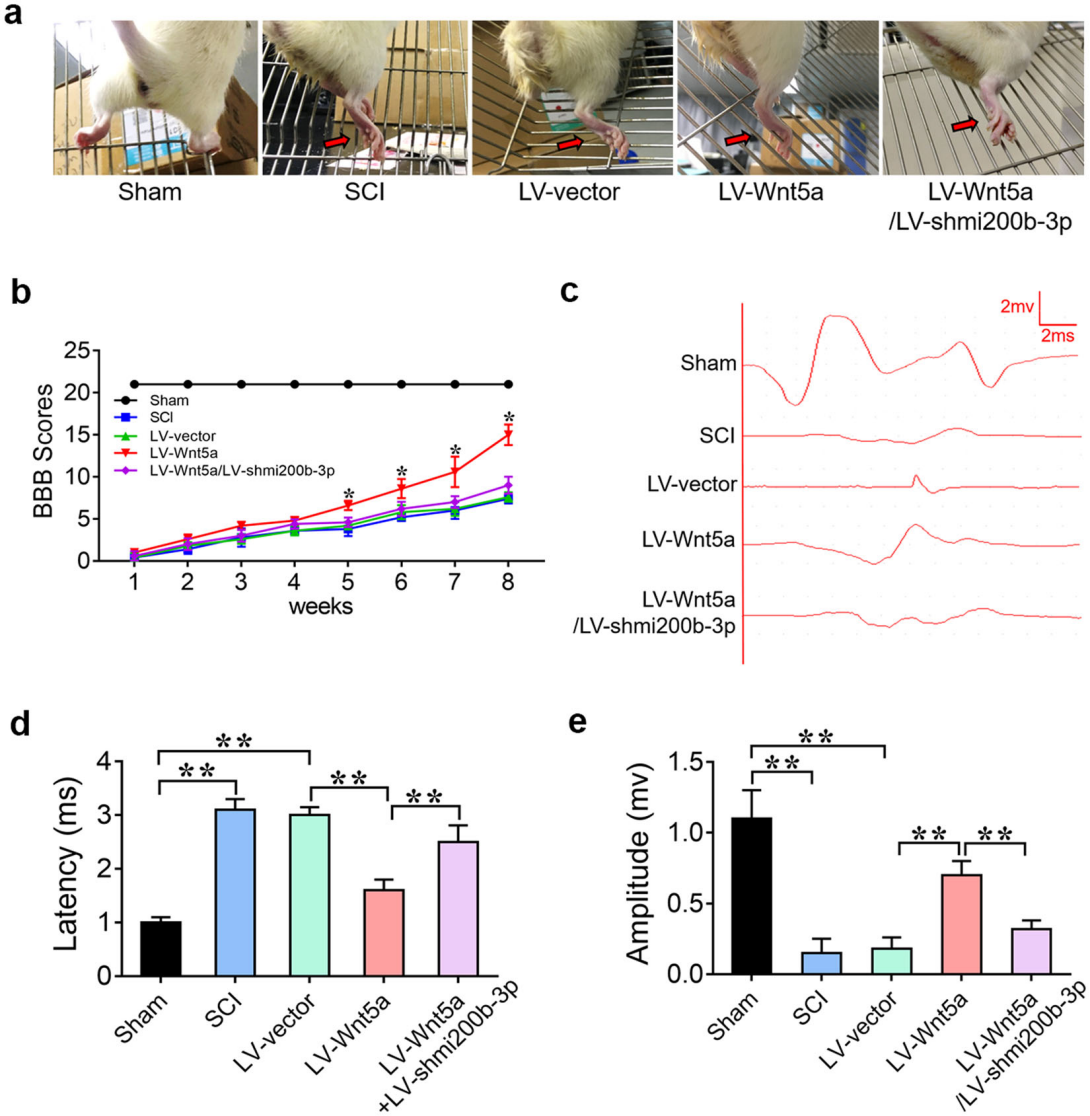
Wnt5a-modified NSCs promote locomotor functional recovery in SCI. **a** Behavioral character images showing hindlimb movement in different groups. **b** BBB scores in different groups. **c** Electrophysiological outcomes of the SCEP in different groups. **d** and **e** Quantification of the SCEP latency and amplitude. (**P < 0.01 compared between groups.).

To investigate whether Wnt5a can enhance the therapeutic benefit of NSC transplantation in vivo. LV-Wnt5a- and LV-shmi200b-3p-transfected NSCs were injected into the injury site to assess the effect of LV-Wnt5a-transfected NSC transplantation on functional recovery. As shown in Fig. 4, the rats in the sham group could grab and step easily using the hindlimb, whereas the rats in the SCI group could hardly grab or stand up. As expected, the rats in the LV-Wnt5a group could grab mildly and step slowly using their hindlimb, whereas the rats in the LV-Wnt5a/LV-shmi200b-3p group could hardly grab or stand up, like those in the SCI group (Fig. 4a). To determine whether behavioral function had been repaired, we used the BBB score to evaluate hindlimb locomotor activity after SCI, and as expected, hindlimb locomotion was zero immediately after the operation for all rats. Over the course of 2 months, the rats in the LV-Wnt5a group exhibited significantly higher BBB scores than those in the SCI group; however, the rats in the LV-Wnt5a/LV-shmi200b-3p group exhibited the same BBB scores as those in the SCI group (Fig. 4b). To further confirm sensory and motor functional recovery, we performed electrophysiological analysis. The SCEP in the LV-Wnt5a group was stronger, the latency was shorter, and the amplitude was higher than that in the SCI and LV-vector groups. However, the SCEP in the LV-Wnt5a/LV-shmi200b-3p group was weaker, the latency was longer, and the amplitude was lower than that in the LV-Wnt5a group (Fig. 4c–e). These results suggest that Wnt5a promoted motor functional recovery after spinal cord injury in an mi200b-3p-dependent manner.

**Fig. 5:**
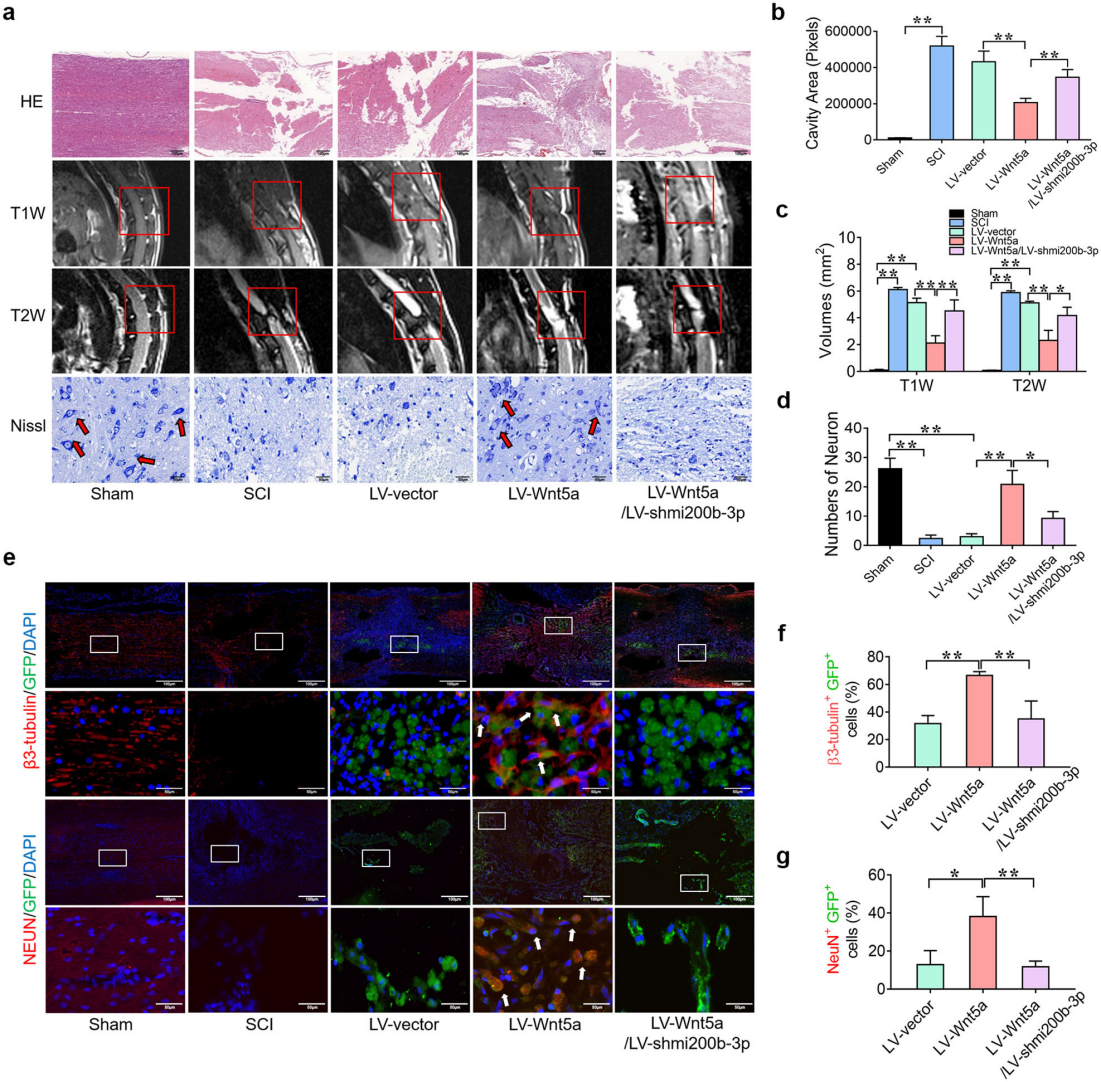
Wnt5a-modified NSCs promote histological repair of the damaged spinal cord in SCI. **a** H&E staining, Nissl staining, and MRI analyses of the spinal cord in different groups. **b**–**d** Quantification of H&E staining, Nissl staining and MRI results in different groups. **e** Immunofluorescence staining of the spinal cord in different groups. The white boxes indicate images of higher magnification. The white arrow indicates colocalized cells. **f** and **g** Quantification of immunofluorescence staining. (*P < 0.05 compared between groups; **P < 0.01 compared between groups.).

To further determine the tissue repair effect of Wnt5a-NSC transplantation in vivo, the lesion cavity was calculated on H&E staining at 8 weeks after the operation to detect tissue repair. The total size of the lesion cavity in the LV-Wnt5a group was significantly smaller than that in the SCI group. However, the total size of the lesion cavity in the LV-Wnt5a/LV-shmi200b-3p group was larger than that in the LV-Wnt5a group (Fig. 5a, b). We also used MRI to measure the volume of the injured spinal cord. The volume of the injured spinal cord cavity was identified as a hypointense region in T1-weighted images and a hyperintense region in T2-weighted images. Findings similar to those of H&E staining were observed on MRI (Fig. 5a, c). Furthermore, the number of ventral horn motor neurons at the lesion epicenter was calculated using Nissl staining. The number of surviving neurons in the LV-Wnt5a group was significantly increased compared with that in the SCI group and significantly decreased in the LV-Wnt5a/LV-shmi200b-3p group compared with that in the LV-Wnt5a group (Fig. 5a, d). We further investigated the differentiation status of the transplanted NSCs around the injured site of the spinal cord. In the LV-vector and LV-Wnt5a/LV-shmi200b-3p groups, only a few GFP-positive NSCs showed early neuronal marker (β3-tubulin) and mature neuronal marker (NeuN) expression. In contrast, the number of GFP^+^ β3-tubulin^+^ cells and GFP^+^ NeuN^+^ cells was significantly increased in the LV-Wnt5a group (Fig. 5e–g). In addition, our results showed that some GFP-positive NSCs expressed an oligodendrocyte marker (MBP) in the LV-Wnt5a group, and only a few GFP-positive NSCs expressed an astrocyte marker (GFAP) in the LV-Wnt5a group. In the LV-vector and LV-Wnt5a/LV-shmi200b-3p groups, the number of GFP^+^ MBP^+^ cells was significantly decreased, and the number of GFP^+^ GFAP^+^ cells was significantly increased (Supplementary Fig. S3a–c). These results suggest that Wnt5a induced transplanted NSCs to mainly differentiate into neurons through upregulated mi200b-3p expression to promote spinal cord repair after SCI.

### Wnt5a induces NSC differentiation into neurons by suppressing RhoA expression to promote spinal cord repair in SCI

**Fig. 6:**
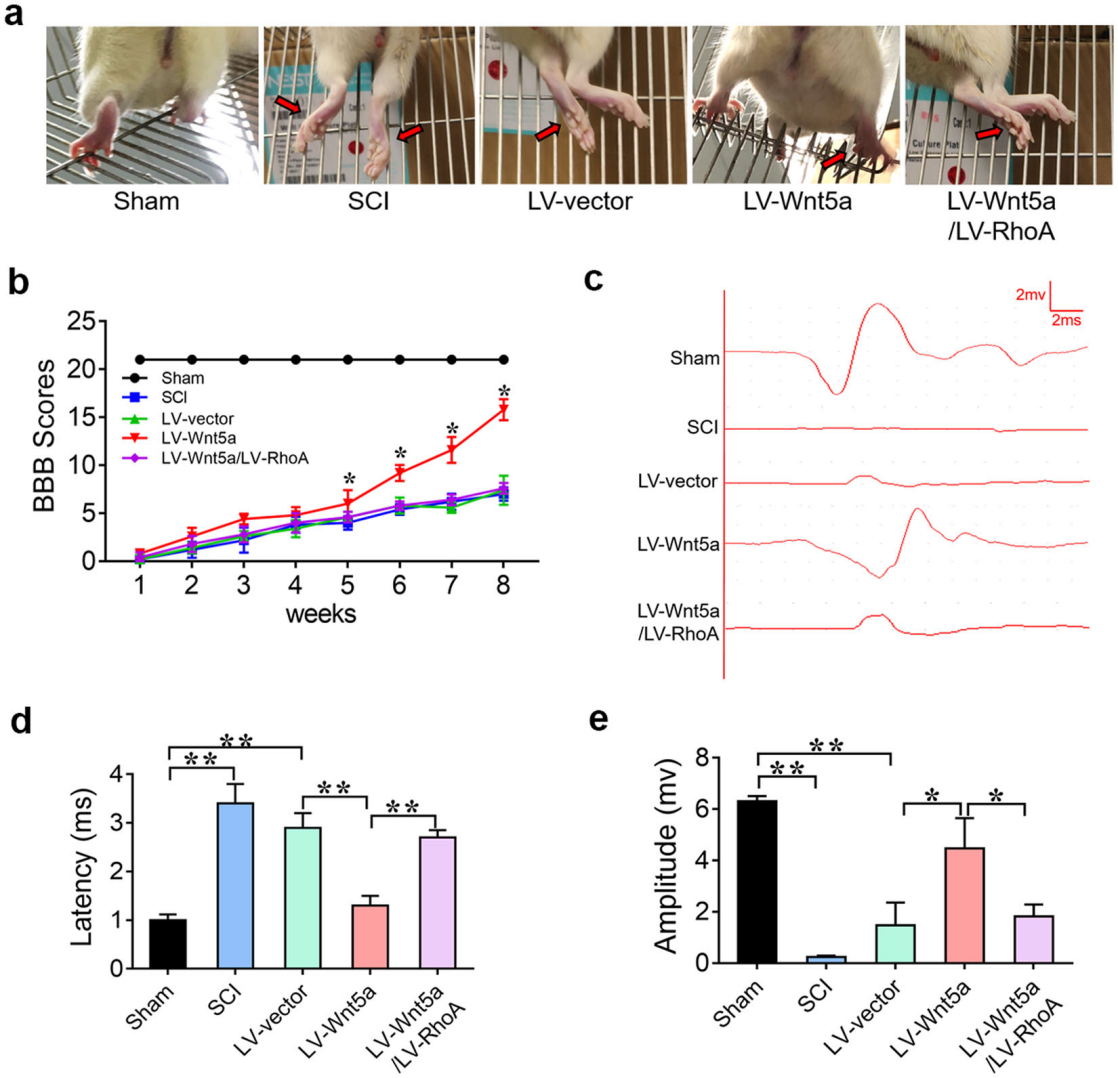
Wnt5a induces neuronal differentiation by suppressing RhoA expression to promote locomotor functional recovery in SCI. **a** Behavioral character images showing hindlimb movement in different groups. **b** BBB scores in different groups. **c** Electrophysiological outcomes of the SCEP in different groups. **d** and **e** Quantification of SCEP latency and amplitude. (*P < 0.05 compared between groups; **P < 0.01 compared between groups.).

To further determine whether Wnt5a promotes spinal cord recovery by suppressing RhoA expression in vivo, LV-Wnt5a- and LV-RhoA-transfected NSCs were injected into the injury site to assess the effect on functional recovery. As expected, the rats in the LV-Wnt5a group could grab mildly and step slowly using their hindlimb, whereas the rats in the LV-Wnt5a/LV-RhoA group could hardly grab or stand up, like those in the SCI group (Fig. 6a). BBB and electrophysiological analyses showed that rats in the LV-Wnt5a group exhibited better motor functional recovery, while rats in the LV-Wnt5a/LV-RhoA group exhibited poor motor functional recovery after SCI (Fig. 6b–e). These results suggest that Wnt5a promotes motor functional recovery in SCI by suppressing RhoA expression.

**Fig. 7:**
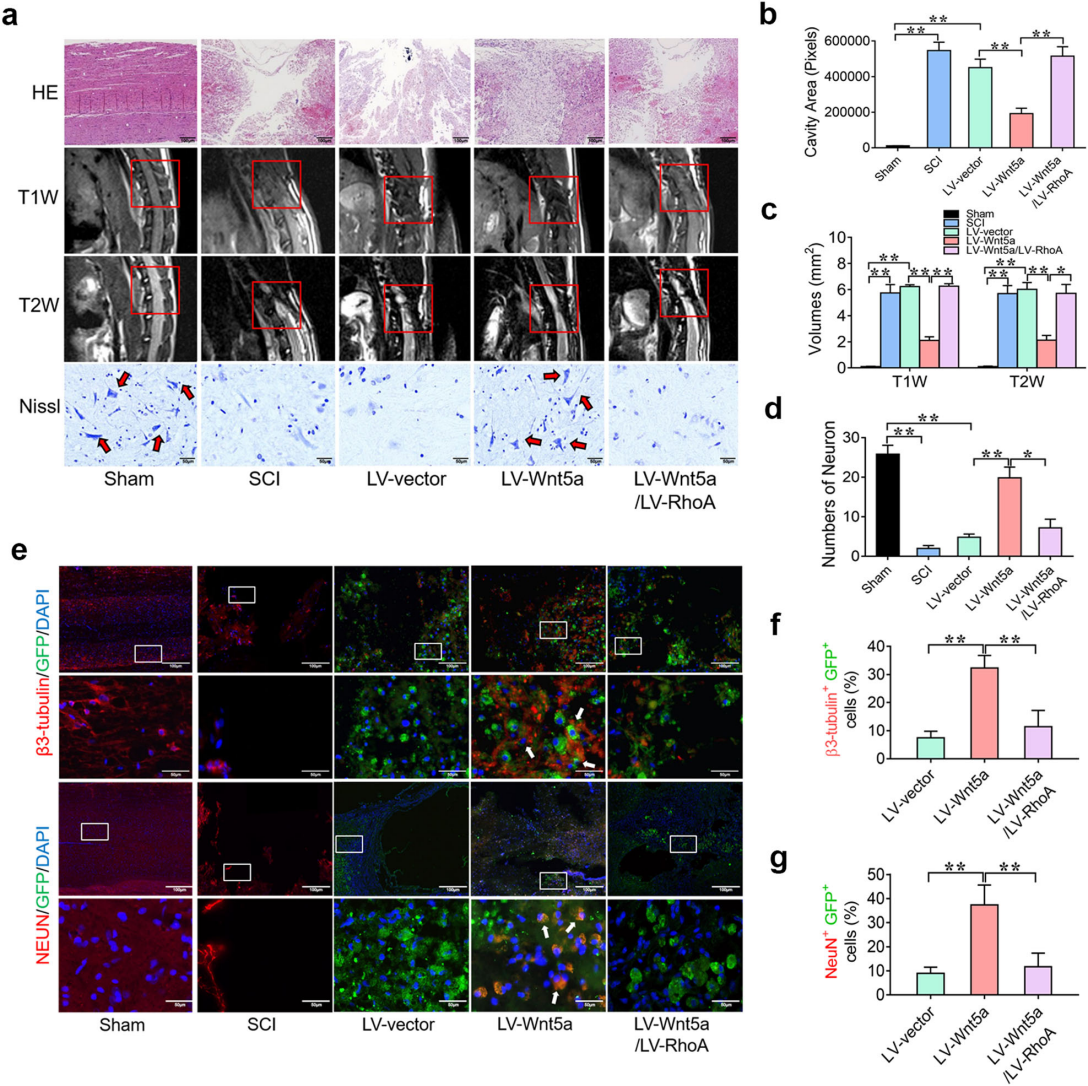
Wnt5a induces neuronal differentiation by suppressing RhoA to promote histological repair of the damaged spinal cord in SCI. **a** H&E staining, Nissl staining and MRI analyses of the spinal cord in different groups. **b**–**d** Quantification of H&E staining, Nissl staining and MRI results in different groups. **e** Immunofluorescence staining of the spinal cord in different groups. The white boxes indicate images of higher magnification. The white arrow indicates colocalized cells. **f** and **g** Quantification of immunofluorescence staining results. (*P < 0.05 compared between groups; **P < 0.01 compared between groups.).

To further determine the tissue repair effect of Wnt5a through suppressing RhoA expression in vivo, H&E staining, MRI and Nissl staining were used to detect tissue repair at 8 weeks after the operation. The total size of the lesion cavity in the LV-Wnt5a group was significantly smaller than that in the SCI group; however, the total size of the lesion cavity in the LV-Wnt5a/LV-RhoA group was larger than that in the LV-Wnt5a group (Fig. 7a, b). Findings similar to those of H&E staining were observed on MRI (Fig. 7a, c). Nissl staining showed that the number of surviving neurons in the LV-Wnt5a group was significantly increased compared with that in the SCI group. The number of surviving neurons in the LV-Wnt5a/LV-RhoA group was significantly decreased compared with that in the LV-Wnt5a group (Fig. 7a, d). Furthermore, we investigated the differentiation status of the transplanted NSCs around the injured site of the spinal cord. In the LV-vector group and the LV-Wnt5a/LV-RhoA group, only a few GFP-positive cells showed early neuronal marker (β3-tubulin) and mature neuronal marker (NeuN) expression. In contrast, the number of GFP^+^ β3-tubulin^+^ cells and GFP^+^ NeuN^+^ cells was significantly increased in the LV-Wnt5a group (Fig. 7e–g). In addition, the number of GFP^+^ MBP^+^ cells was significantly decreased and the number of GFP^+^ GFAP^+^ cells was significantly increased in the LV-Wnt5a/LV-RhoA groups (Supplementary Fig. S3a–c). These results suggest that Wnt5a induced NSCs to mainly differentiate into neurons by suppressing RhoA expression to promote spinal cord repair after SCI.

## Discussion

Spinal cord injury due to external trauma is a difficult clinical problem without an ideal solution thus far^2,35^. Currently, the current therapeutic strategies for SCI are not sufficient due to the failure of axonal and neural regeneration^36^. Thus, an agent that is able to suppress negative effects on neuronal differentiation and simultaneously promote neurogenesis in the microenvironment would be of great benefit for the treatment of traumatic spinal cord injury. Since Wnt signaling has been proven to be neurogenic, we speculated that Wnt5a might have therapeutic potential in traumatic spinal cord injury.

In the current study, we recorded three key observations that provide insights into Wnt5a-induced neurogenesis. First, we confirmed that Wnt5a induced neuronal differentiation and suppressed RhoA/Rock signaling in NSCs. Second, we provided the first evidence that mi200b-3p was upregulated by Wnt5a and essential for both the neurogenesis and suppression of RhoA signaling mediated by Wnt5a. Third, we provided in vivo evidence that mi200b-3p is critical for Wnt5a to induce transplanted NSCs to differentiate into neurons to promote functional and histological recovery by suppressing RhoA signaling after SCI.

Previous studies have shown that Wnt5a has a neurogenic effect. Several classic pathways have been found to be involved in neuronal differentiation. Varela-Nallar et al.^37^ reported that Wnt5a signaling via the Wnt/Ca+ pathway stimulated dendritic spine morphogenesis in hippocampal neurons and played a trophic role in neuronal differentiation. Wnt5a also activated the JNK and Rac1 pathways to promote ventral midbrain morphogenesis and dopaminergic differentiation^38^. However, there is substantial evidence that activating noncanonical pathways suppresses axonal and neural growth. Wnt-Ryk pathways have been reported to suppress axonal and neurite growth^39,40^. Elevating the expression of noncanonical Wnt ligands contributes to the lack of axonal regeneration in CNS models, while blocking noncanonical Wnt signaling promotes axonal growth and functional recovery^39,41,42^. In addition, miRNAs have been reported to play critical roles in regulating gene expression at the posttranscriptional level in neural development, tumor metastasis, and cell proliferation, apoptosis and differentiation^24^. Previous studies have reported that RhoA, which was recognized to suppress neuronal differentiation^26,43^, was the target gene of mi200b-3p and that RhoA expression was downregulated by mi200b-3p^25^.

In this study, we confirmed that Wnt5a, which has been controversial in neurogenesis, to the best of our knowledge, and mi200b-3p have strong positive effects on neurogenesis in NSCs (Supplementary Figs. S1 and S2). In addition, we provided evidence that the expression levels of both Wnt5a and mi200b-3p were elevated in the process of neuronal differentiation, mi200b-3p was upregulated by Wnt5a through the MAPK/JNK pathway in NSCs, and mi200b-3p played a positive role in neurogenesis. The inhibition of mi200b-3p partially but significantly suppressed Wnt5a-induced neuronal differentiation. These results strongly suggest that Wnt5a upregulated mi200b-3p expression through the MAPK/JNK pathway to promote neuronal differentiation (Fig. 1).

The underlying mechanism of Wnt5a-induced neuronal differentiation is not yet clear. Previous studies have reported a negative effect of RhoA/Rock signaling on neuronal differentiation. Noncanonical Wnt-planar cell polarity (PCP) signaling controls Rho GTPase activity locally by activating or suppressing RhoA and Rac1, resulting in many biological processes^44,45^. Yang et al.^26^ demonstrated that Syx is a gene encoding a RhoA-specific guanine nucleotide exchange factor. Noggin and RARγ, which are proteins involved in neural differentiation, were more abundant in embryonic Syx^-/-^ cells. These phenomena were blocked by the overexpression of active RhoA. This strongly suggested that RhoA/Rock signaling prevented neuronal differentiation by limiting the promotion of neuronal differentiation protein expression. Our results reveal that Wnt5a significantly suppressed RhoA and Rock1 expression during neuronal differentiation. RhoA is the target gene of mi200b-3p according to a biological database, and with mi200b-3p silencing, the suppressive effect of Wnt5a was abolished (Fig. 2). These results indicate that mi200b-3p not only mediates the direct effect of Wnt5a on promoting neurogenesis but also the inhibitory effect of Wnt5a on the RhoA pathway. Moreover, our results show that the overexpression of RhoA could resist the neurogenic effect of Wnt5a and mi200b-3p in NSCs. All the results suggest that Wnt5a/mi200b-3p promoted neuronal differentiation by suppressing activation of the RhoA pathway (Fig. 3).

The transplantation of NSCs is considered to be a potential therapeutic strategy in SCI because NSCs can differentiate into neurons and oligodendrocytes to reconnect the neural circuit in the lesion^1,46^. However, previous studies have reported that most NSCs transplanted into SCI lesions differentiate into astrocytes rather than neurons^11^. Thus, an agent that increases the neuronal differentiation rate of NSCs is necessary for NSC transplantation therapy in SCI. To confirm the role of Wnt5a and mi200b-3p in neuronal differentiation and regeneration, we further conducted an in vivo experiment. Behavioral characterization, BBB score evaluation and SCEP analyses revealed that the transplantation of Wnt5a-transfected NSCs could lead to better locomotor recovery than naive NSC transplantation. The silencing of mi200b-3p in Wnt5a-transfected NSCs significantly suppressed locomotor recovery in rodents with SCI (Fig. 4). Moreover, our histological results revealed that the transplantation of Wnt5a-transfected NSCs could lead to better spinal cord tissue repair than the transplantation of naive NSCs, and the silencing of mi200b-3p in Wnt5a-transfected NSCs was found to significantly abolish the recovery effect. Most Wnt5a-transfected NSCs could differentiate into neurons rather than astrocytes, the latter of which are disadvantageous in spinal cord repair, to reconnect the neural circuit, and this phenomenon required the involvement of mi200b-3p (Fig. 5 and Supplementary Fig. S3).

Finally, we further confirmed that Wnt5a/mi200b-3p improved locomotor functional recovery and promoted tissue repair by suppressing the activation of RhoA signaling. Behavioral characterization, BBB score evaluation and SCEP analyses revealed that the transplantation of Wnt5a-transfected NSCs could lead to better locomotor recovery than the transplantation of naive NSCs, and the overexpression of RhoA in Wnt5a-transfected NSCs was found to sabotage the therapeutic effect of Wnt5a-transfected NSCs in rodents with SCI (Fig. 6). Moreover, our histological results revealed that the transplantation of Wnt5a-transfected NSCs could lead to better spinal cord tissue repair than the transplantation of naive NSCs, and the overexpression of RhoA in Wnt5a-transfected NSCs was found to significantly abolish the recovery effect. Most Wnt5a-transfected NSCs could differentiate into neurons to reconnect the neural circuit, and this phenomenon was inhibited by the overexpression of RhoA (Fig. 7 and Supplementary Fig. S3). In addition, some Wnt5a-transfected NSCs were found to differentiate into oligodendrocytes, which are also beneficial for spinal cord repair^47,48^. The overexpression of RhoA induced NSCs to mainly differentiate into astrocytes. This result indicates that Wnt5a have potential as a therapeutic agent to optimize NSC transplantation after SCI.

**Fig. 8:**
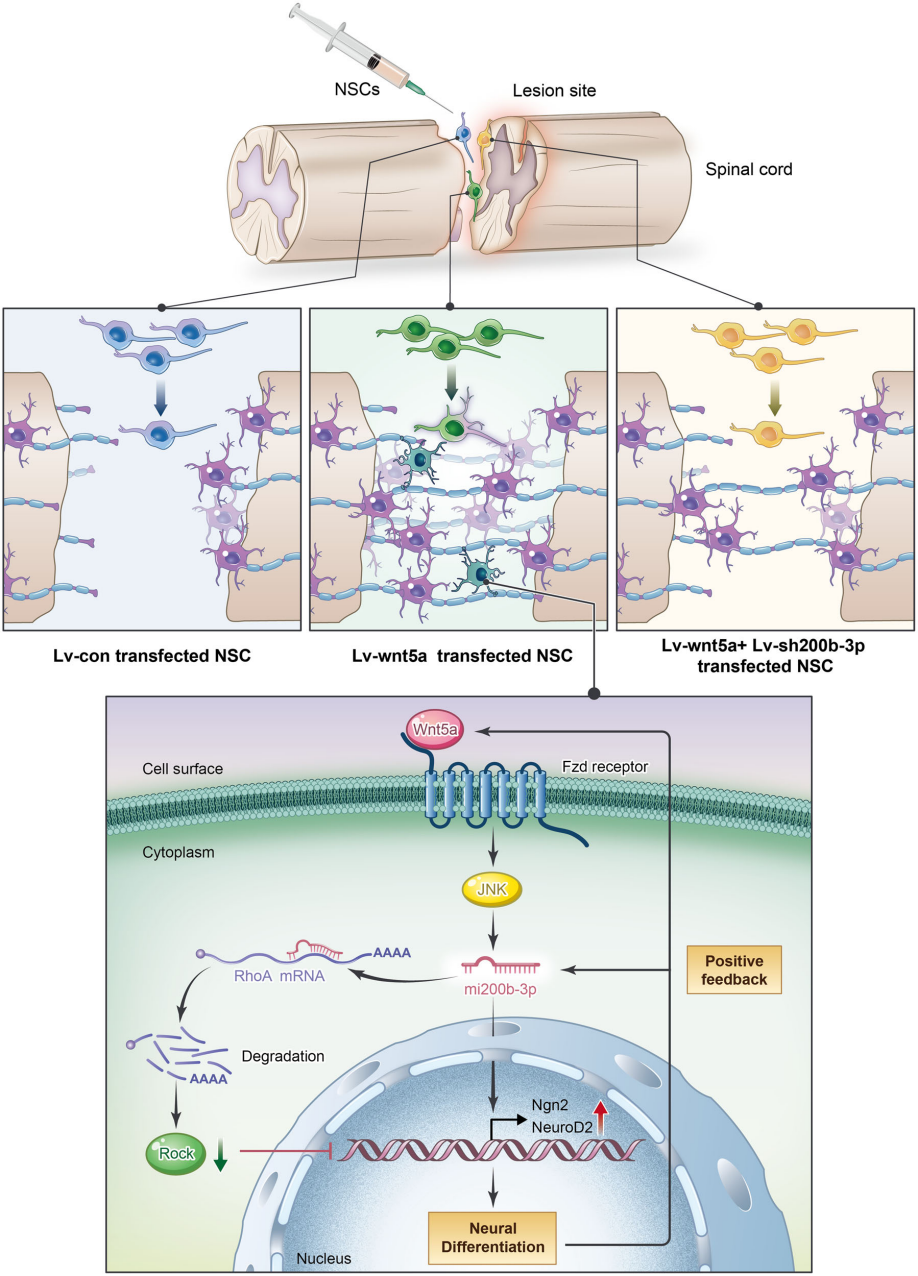
A schematic diagram illustrating the theory in the current study. Wnt5a-transfected NSCs were injected into the injury site to assess the therapeutic effect of Wnt5a-modified NSC transplantation on functional recovery. miRNA200b-3p plays a critical role in Wnt5a promoting neurogenesis and suppressing the RhoA/Rock1 signaling pathway.

In conclusion, we showed a novel mechanism of Wnt5a-induced neuronal differentiation. Wnt5a-induced miRNA200b-3p expression is essential for the neuroinductive effect and the inhibitory effect of the RhoA/Rock pathway on neuronal differentiation in spinal cord injury (Fig. 8).

## Supplementary information

Supplementary Materials (Clean version)

## References

[1] Assinck P, Duncan GJ, Hilton BJ, Plemel JR, Tetzlaff W (2017). Cell transplantation therapy for spinal cord injury. Nat. Neurosci..

[2] Ahuja CS (2017). Traumatic spinal cord injury-repair and regeneration. Neurosurgery.

[3] Lee BB, Cripps RA, Fitzharris M, Wing PC (2014). The global map for traumatic spinal cord injury epidemiology: update 2011, global incidence rate. Spinal Cord..

[4] Jin, M. C., Medress, Z. A., Azad, T. D., Doulames, V. M. & Veeravagu, A. Stem cell therapies for acute spinal cord injury in humans: a review. *Neurosurg. Focus* **46**, Artn E10 10.3171/2018.12.Focus18602 (2019). 10.3171/2018.12.FOCUS18602 30835679

[5] Gomes ED, Silva NA, Salgado AJ (2019). Combinatorial therapies for spinal cord injury: strategies to induce regeneration. Neural Regen. Res.

[6] Mackay-Sim A, St John JA (2011). Olfactory ensheathing cells from the nose: Clinical application in human spinal cord injuries. Exp. Neurol..

[7] Saberi H (2011). Safety of intramedullary Schwann cell transplantation for postrehabilitation spinal cord injuries: 2-year follow-up of 33 cases. J. Neurosurg.-Spine.

[8] Ogawa Y (2002). Transplantation of in vitro-expanded fetal neural progenitor cells results in neurogenesis and functional recovery after spinal cord contusion injury in adult rats. J. Neurosci. Res..

[9] Lee KZ (2014). Intraspinal transplantation and modulation of donor neuron electrophysiological activity. Exp. Neurol..

[10] Zhu YC, Uezono N, Yasui T, Nakashima K (2018). Neural stem cell therapy aiming at better functional recovery after spinal cord injury. Dev. Dynam.

[11] Klein S, Svendsen CN (2005). Stem cells in the injured spinal cord: reducing the pain and increasing the gain. Nat. Neurosci..

[12] Qu QH (2010). Orphan nuclear receptor TLX activates Wnt/beta-catenin signalling to stimulate neural stem cell proliferation and self-renewal. Nat. Cell Biol..

[13] Rodriguez JP (2014). Abrogation of beta-catenin signaling in oligodendrocyte precursor cells reduces glial scarring and promotes axon regeneration after CNS injury. J. Neurosci..

[14] Patel, M. et al. Prolonged neural stem cell maturation restores motor function in spinal cord-lesioned rats. *Nat. Rev. Neurol.* **13**, 10.1038/nrneurol.2017.133 (2017). 10.1038/nrneurol.2017.133 28914887

[15] Seitz R, Hackl S, Seibuchner T, Tamm ER, Ohlmann A (2010). Norrin mediates neuroprotective effects on retinal ganglion cells via activation of the Wnt/beta-catenin signaling pathway and the induction of neuroprotective growth factors in muller cells. J. Neurosci..

[16] Jung YS (2018). TMEM9 promotes intestinal tumorigenesis through vacuolar-ATPase-activated Wnt/beta-catenin signalling. Nat. Cell Biol..

[17] Li, M. W. et al. Transmembrane protein 170B is a novel breast tumorigenesis suppressor gene that inhibits the Wnt/beta-catenin pathway. *Cell Death Dis.* **9**, ARTN 91 10.1038/s41419-017-0128-y (2018). 10.1038/s41419-017-0128-y PMC5833782 29367600

[18] Bresson, L. et al. Podoplanin regulates mammary stem cell function and tumorigenesis by potentiating Wnt/beta-catenin signaling. *Development* **145**, doi:UNSP dev160382 10.1242/dev.160382 (2018). 10.1242/dev.160382 29361573

[19] Hirabayashi Y (2004). The Wnt/beta-catenin pathway directs neuronal differentiation of cortical neural precursor cells. Development.

[20] Kuwabara T (2009). Wnt-mediated activation of NeuroD1 and retro-elements during adult neurogenesis. Nat. Neurosci..

[21] Park, S. Y., Kang, M. J. & Han, J. S. Interleukin-1 beta promotes neuronal differentiation through the Wnt5a/RhoA/ JNK pathway in cortical neural precursor cells. *Mol Brain* **11**, ARTN 39 10.1186/s13041-018-0383-6 (2018). 10.1186/s13041-018-0383-6 PMC6033214 29973222

[22] Jang, S., Park, J. S. & Jeong, H. S. Neural differentiation of human adipose tissue-derived stem cells involves activation of the Wnt5a/JNK signalling. *Stem Cells Int*. **2015**, Artn 178618 10.1155/2015/178618 (2015). 10.1155/2015/178618 PMC4461786 26106419

[23] Blakely BD (2013). Ryk, a receptor regulating Wnt5a-mediated neurogenesis and axon morphogenesis of ventral midbrain dopaminergic neurons. Stem Cells Dev..

[24] Smirnova L (2005). Regulation of miRNA expression during neural cell specification. Eur. J. Neurosci..

[25] Ma, T. & Xue, Y. X. MiRNA-200b regulates RMP7-induced increases in blood-tumor barrier permeability by targeting RhoA and ROCKII. *Front. Mol. Neurosci*. **9**, doi:ARTN 9 10.3389/fnmol.2016.00009 (2016). 10.3389/fnmol.2016.00009 PMC4742559 26903801

[26] Yang, J. N. et al. RhoA inhibits neural differentiation in murine stem cells through multiple mechanisms. *Sci. Signal.* **9**, doi:ARTN ra76 10.1126/scisignal.aaf0791 (2016). 10.1126/scisignal.aaf0791 PMC5241026 27460990

[27] Chen NN (2016). Targeted inhibition of leucine-rich repeat and immunoglobulin domain-containing protein 1 in transplanted neural stem cells promotes neuronal differentiation and functional recovery in rats subjected to spinal cord injury. Crit. Care Med..

[28] Zhao, X. Y. et al. Lentiviral vector delivery of short hairpin RNA to NgR1 promotes nerve regeneration and locomotor recovery in injured rat spinal cord. *Sci. Rep-Uk* **8**, doi:Artn 5447 10.1038/S41598-018-23751-2 (2018). 10.1038/s41598-018-23751-2 PMC5882972 29615686

[29] Li X (2020). Wnt4-modified NSC transplantation promotes functional recovery after spinal cord injury. Faseb J..

[30] Kanekiyo K (2017). Effects of multiple injection of bone marrow mononuclear cells on spinal cord injury of rats. J. Neurotraum.

[31] Wu HF (2013). The promotion of functional recovery and nerve regeneration after spinal cord injury by lentiviral vectors encoding Lingo-1 shRNA delivered by Pluronic F-127. Biomaterials.

[32] Simard JM (2013). MRI evidence that glibenclamide reduces acute lesion expansion in a rat model of spinal cord injury. Spinal Cord..

[33] Ohta K, Fujimura Y, Nakamura M, Watanabe M, Yato Y (1999). Experimental study on MRI evaluation of the course of cervical spinal cord injury. Spinal Cord..

[34] Simon CM, Vaughan EM, Bement WM, Edelstein-Keshet L (2013). Pattern formation of Rho GTPases in single cell wound healing. Mol. Biol. Cell.

[35] Gabel BC, Curtis EI, Marsala M, Ciacci JD (2017). A review of stem cell therapy for spinal cord injury: large animal models and the frontier in humans. World Neurosurg..

[36] Binan L, Ajji A, De Crescenzo G, Jolicoeur M (2014). Approaches for neural tissue regeneration. Stem Cell Rev. Rep..

[37] Varela-Nallar L, Aranguiz FC, Abbott AC, Slater PG, Inestrosa NC (2010). Adult hippocampal neurogenesis in aging and Alzheimer’s Disease. Birth Defects Res. C..

[38] Andersson ER (2013). Wnt5a cooperates with canonical Wnts to generate midbrain dopaminergic neurons in vivo and in stem cells. Proc Natl Acad. Sci. USA.

[39] Tury A, Tolentino K, Zou YM (2014). Altered expression of atypical PKC and Ryk in the spinal cord of a mouse model of amyotrophic lateral sclerosis. Dev. Neurobiol..

[40] Lanoue, V. et al. The Wnt receptor Ryk is a negative regulator of mammalian dendrite morphogenesis. *Sci. Rep-Uk* **7**, doi:Artn 5965 10.1038/S41598-017-06140-Z (2017). 10.1038/s41598-017-06140-z PMC5519545 28729735

[41] Salinas, P. C. Wnt signaling in the vertebrate central nervous system: from axon guidance to synaptic function. *Cold Spring Harb. Perspect. Biol*. **4**, doi:ARTN a008003 10.1101/cshperspect.a008003 (2012). 10.1101/cshperspect.a008003 PMC3281574 22300976

[42] Garcia AL, Udeh A, Kalahasty K, Hackam AS (2018). A growing field: the regulation of axonal regeneration by Wnt signaling. Neural Regen. Res..

[43] Matsukawa T, Morita K, Omizu S, Kato S, Koriyama Y (2018). Mechanisms of RhoA inactivation and CDC42 and Rac1 activation during zebrafish optic nerve regeneration. Neurochem. Int.

[44] Gao C, Chen YG (2010). Dishevelled: the hub of Wnt signaling. Cell Signal.

[45] Mayor R, Theveneau E (2014). The role of the non-canonical Wnt-planar cell polarity pathway in neural crest migration. Biochem. J..

[46] Tang, Y. W., Yu, P. & Cheng, L. Current progress in the derivation and therapeutic application of neural stem cells. *Cell Death Dis.* **8**, doi:Artn E3108 10.1038/Cddis.2017.504 (2017). 10.1038/cddis.2017.504 PMC5682670 29022921

[47] Almad A, Sahinkaya FR, McTigue DM (2011). Oligodendrocyte fate after spinal cord injury. Neurotherapeutics.

[48] Alizadeh A, Karimi-Abdolrezaee S (2016). Microenvironmental regulation of oligodendrocyte replacement and remyelination in spinal cord injury. J. Physiol.-Lond..

