# Supplementary material for: Transplantation of Wnt5a-modified NSCs promotes tissue repair and locomotor functional recovery after spinal cord injury
Source: Exp Mol Med. 2020 Dec 14;52(12):2020–33. doi: 10.1038/s12276-020-00536-0 (PMC8080632; doi:10.1038/s12276-020-00536-0)
Supplement: Supplementary file 1 — Supplementary Materials (Clean version) [file 12276_2020_536_MOESM1_ESM.doc]

**Supplementary Materials**

Materials and Methods in detail

Supplementary Reference

Supplementary Fig. 1. Wnt5a promotes neuronal differentiation in NSC

Supplementary Fig. 2. miRNA200b-3p promotes neuronal differentiation in NSC

Supplementary Fig. 3. The differentiation status of the transplanted NSC at the injured site after SCI

Supplementary Table 1. Primers for qRT-PCR analysis of gene expression

**Materials and Methods in detail**

**NSC Isolation, Culturing, and Transfection**

All procedures performed on experimental animals were approved by the Animal Care and Use Committee of Sun Yat-sen University and were conducted in accordance with the Guide to the Care and Use of Experimental Animals by the National Research Council (1996, United States).

NSC cultures were obtained from the fetal brains of embryonic day 14 rats, which were extracted from pregnant Sprague-Dawley (SD) rats (Laboratory Animal Center of Sun Yat-sen University, Guangzhou, China) ^1-3^. Briefly, the brain tissue was mechanically dissected and dissociated in Hanks Balanced Salt Solution, and the cell suspension was centrifuged at 1,000 rpm for 5 minutes. The supernatant was discarded, and the cell pellet was diluted to a single-cell suspension. NSC were plated on a T25 culture flask (Corning, Acton, MA, 430639) containing maintenance medium which was consisted of Dulbecco Modified Eagle Medium/F-12 nutrient mixture, 2% B27, 1% penicillin/streptomycin, 1% l-glutamine (Invitrogen, Carlsbad, CA, 11320033), 20 ng/mL fibroblast growth factor-2 (FGF-2) and 20 ng/mL epidermal growth factor (EGF) (Peprotech, Rocky Hill, NJ, 96-400-29, 96-AF-100-15). NSC were cultured at 37°C in 5% CO_2_ and were passaged via weekly digestion with Accutase (Millipore, Bedford, MA, SCR005) in the medium described above. All NSC used in this study were between passages 2 and 4.

To induce neural differentiation, cells were plated at a density of 2x10^5^ cells/well in 6- or 12-well tissue-culture plates and allowed to adhere for 24 h at 37°C, at which time cells were switch to neural differentiated medium consisting of basic medium supplemented with 2% B27, 1% penicillin/streptomycin, 1% l-glutamine. The medium was changed every 2-3 days ^3^.

**Lentiviral Vector Construction**

Lentiviruses carrying green fluorescent protein (GFP), Wnt5a gene (NM_022631) overexpression, RhoA gene (NM_057132) overexpression and mi200b-3p gene silencing were constructed as previously described ^4,5^. Briefly, all the full-length Rat genes were encoded into different vectors. The vectors and corresponding packaging plasmids were co-transfected into 293 T cells using Lipofectamine 2000 (Invitrogen, Carlsbad, CA, 11668500). The medium was changed with complete medium after 8 hours of incubation. After 48 hours, the supernatant was harvested from 293 T cells, filtered using a 0.45-μm pore size filter, and concentrated via ultracentrifugation at 96,500 g for 2 hours at 4 °C. After resuspension, the serially diluted lentivirus was used to transduce 293 T cells. Then, 4 days later, the labeled 293 T cells were counted to calculate the viral titer, and high-titer recombinant lentiviral vectors carrying Wnt5a, RhoA and short hairpin mi200b-3p were harvested. The sequence for the specifically silences the mi200b-3p was as follow: Sense: 5’-TAATACTGCCTGGTAATGATGAC GTCATCATTACCAGGCAGTATTA-3’. The final lentiviral vector concentrations of Wnt5a and RhoA were 2x10^9^ TU/mL; lentiviral vector concentrations of shmi200b-3p was 6x10^8^ TU/ mL.

**Transduction**

Cells in log phase were plated at a concentration of 1x10^5^ cells/ well in 6-well plates and transduced with the control lentiviruses, Wnt5a-overexpressing lentivirus, RhoA-overexpressing lentivirus (LV-vector, LV-Wnt5a and LV-RhoA) and short hairpin mi200b-3p lentiviruses (LV-shmi200b-3p) in MEM-α with 10% FBS. Polybrene (Sigma-Aldrich, MO, TR-1003) was added as an enhancing reagent to improve transduction efficiency, at a concentration of 10 mg/ml. After 8 hours, the medium was changed with fresh complete medium. Cells were harvested for injection of Spinal cord injury model after transduction, respectively.

**Transfection of microRNA mimic and inhibitor**

NSC were digested using trypsin and re-suspended by differentiation medium. The cells were incubated on 6-well plate, 2ml suspension was added per well. 15ul Polo3000 (Invitrogen, Carlsbad, CA, L3000015) transfection reagent was mixed with 200ul DMEM/F12 medium (Invitrogen, Carlsbad, CA, 11320033), meanwhile 5ul different mimics or inhibitors (Control mimic, mi200b-3p mimic, Control inhibitor and mi200b-3p inhibitors) (GenePharma Co. Ltd. Shanghai, China) was respectively mixed with 200ul DMEM/F12, for 5 min at room temperature. Follow that, mixed the 2 solutions and incubated for 15min, then dripped the transfection reagent mixture into cell suspension ^6^.

**Pharmaceutical inhibition**

The pharmaceutical inhibitor used for cellular experiments were from following source: JNK specific inhibitor (SP600125, Cell Signaling Technology, MA, #8177, 25μM), p38 specific inhibitor (SB202190, Cell Signaling Technology, MA, #8158, 20μM), ERK specific inhibitor (PD98059, Cell Signaling Technology, MA, #9900, 20μM), PKC inhibitor (Rottlerin, R&D, USA, #1610, 10μM).

**Real-time quantitative reverse transcription PCR (RT-qPCR)**

Total RNA was extracted from cells according to the manufacturer’s protocol, and 2 µg of total DNA-free RNA was used to synthesize cDNA using the ReverTra Ace qPCR RT Kit (Toyobo, Osaka, FSQ-101). The reactions were set up in 96-well plates using 1 µl cDNA with Thunderbird SYBR qPCR Mix (Toyobo, Osaka, QPS-201), to which gene-specific forward and reverse PCR primers were added. QRT-PCR was performed under the following conditions: 95°C for 10 min, followed by 40 cycles of 95°C for 10 sec and 55°C for 34 sec. These analyses were performed to detect β3-tubulin, MAP2, GFAP, RhoA and Rock1 expression, and β-actin was used as an internal control. Primer sequences were as Table S1.

**Western blot analysis**

Cells were lyses in RIPA buffer, total protein was extracted, and the protein concentration was determined with a BCA assay. A 10% SDS-PAGE gel was loaded with 20 µg of total protein, and the separated proteins were transferred by electro blotting to PVDF membranes. The membranes were blocked with 5% non-fat dry milk in TBST (50mM Tris, pH 7.6, 150mMNaCl, 0.1% Tween 20) and incubated with the primary antibody overnight at 4°C in 5% non-fat dry milk in TBST. Immunolabelling was detected using ECL reagent (Invitrogen, CA, WP20005). The antibodies used for Western blot were from following source: anti-β3-tubulin antibody (Abcam, UK, ab6161, 1:1000), anti-MAP2 antibody (Abcam, UK, ab11267, 1:1000), anti-GFAP antibody (Abcam, UK, ab7260, 1:1000), anti-RhoA antibody (Abcam, UK, ab187027, 1:1000), anti-Rock1 antibody (Abcam, UK, ab134181, 1:1000), anti-β-actin antibody (Sigma-Aldrich, MO, 1:10000, A1978) and anti-β-tubulin antibody (Sigma-Aldrich, MO, 1:10000, T4026), anti-GAPDH antibody (Sigma-Aldrich, MO, 1:10000, G9545).

**Luciferase reporter assay**

Cells were plated at a density of 1x10^4^ cells/cm^2^ and transfected for 4 hours with a luciferase reporter construct plasmid mixture of 2.9 mg of the luciferase promoter reporter and 0.1 mg of the pGL4.74 Renilla luciferase control vector according to the manufacturer’s instructions. Luciferase activity was measured using the luciferase assay kit (Promega, Madison, WI, E1910) according to the manufacturer’s instructions. The data were normalized to Renilla luciferase activity, and relative luciferase units (RLU) were calculated as firefly luciferase activity/Renilla luciferase activity.

**Immunofluorescence**

Tissue sections from rats (n = 5 per group) and cells (n = 3 per group) were fixed in 4% PFA for 30 minutes and permeabilized with 0.3% Triton X-100 for 30 minutes. Blocking was performed with 5% normal goat serum for 1 hour. The tissue sections and the cells were incubated overnight at 4°C in the primary antibodies against following antigens: anti-β3-tubulin antibody (Abcam, UK, ab6161, 1:200), anti-MAP2 antibody (Abcam, UK, ab11267, 1:200), anti-NeuN antibody (Cell Signaling Technology, MA, #24307, 1:50), anti-MBP antibody (Cell Signaling Technology, MA, #78896, 1:50), anti-GFAP antibody (Cell Signaling Technology, MA, #3670, 1:200). After washing three times in PBS, the primary antibodies were probed with the secondary antibodies Alexa Fluor 594 goat anti-rabbit (Invitrogen, CA, A32740, 1:500) and Alexa Fluor 594 goat anti-mouse (Invitrogen, CA, A21125, 1:500) for 1 hr at room temperature. Finally, the coverslips were washed in PBS three times and mounted using Prolong Gold Antifade Reagent containing 4′-6-diamidino-2-phenylindole (DAPI) (Molecular Probes, Invitrogen, CA, D21490). The targeted marker-positive cells in each visual field were counted under a fluorescence microscope (Carl Zeiss Axio Observer Z1, Zeiss, Oberkochen, Germany).

**Surgical Procedures and Cell Transplantation**

Adult female SD rats (weighing 200-220 g, supplied by the Experimental Animal Center of Sun Yat-sen University, Guangzhou, China) were divided into six groups for this study: Sham group (n = 5), SCI group (n = 5), LV-vector transfected NSC group (n = 5), LV-Wnt5a transfected NSC group (n = 5), LV-Wnt5a/LV-shmi200b-3p co-transfected NSC group (n = 5) and LV-Wnt5a/LV-RhoA co-transfected NSC group (n = 5). After 72 hours of lentiviral transfection, NSC were collected for transplantation. Briefly, animals were anesthetized with 1% Pentobarbital Sodium (40-45mg/kg) and the spinal cord was exposed at the 10th thoracic vertebral level (T10) via laminectomy. The animals in the sham group underwent spinal cord exposure but no injury. In the other five groups, the exposed spinal cord was injured (once rostral to T10 and once caudal to T10) for complete transection. Next, 2 mm of the segmented cord was removed to create a lesion cavity. Once the bleeding was stopped, each group was implanted with 5μl NSC at a density of 1x10^5^ cells/μl to the rostral and caudal of injured site using microsyringe ^1,2^. The T8-T11 spinal cord segments were dissected at 8 weeks after SCI and transplantation to investigate the histological evaluation and differentiated status of the transplanted NSC.

**Functional Assessment**

The 22-point (0-21) Basso, Beattie, and Bresnahan (BBB) open-field locomotor test was used to assess hindlimb locomotor function, including joint movements, stepping ability, coordination, and trunk stability. 21score indicates unimpaired locomotion as observed in sham rats. All animals underwent behavioural testing, and the duration of each session was 5 minutes per rat. Finally, an overall score was calculated ^7^. The evaluation was performed by observers who were blinded to the treatment group of the tested animals.

**Spinal Cord–Evoked Potential Recording (SCEP)**

At 8 weeks postinjury, rats were anesthetized with 1% Pentobarbital Sodium (40-45mg/kg) and stereotaxically fixed. The T5-L1 vertebrae were completely exposed. Briefly, the stimulation electrode was inserted into the T5-T6 interspinous ligaments, and a pair of needle electrodes was inserted into the interspinous ligaments of T12-L1 for spinal cord-evoked potential (SCEP) recording. Then, the electrodes were connected to a BL-410E Data Acquisition Analysis System for Life Science (Chengdu, China). The variables of the SCEP signals were set according to previous reports ^8^ as follows: gain of 2000 time constant of 0.01 seconds, and filtering at 300 Hz. To elicit a SCEP, a single pulse stimulation (50 ms in duration at a frequency of 5.1 Hz and a voltage increase of 1 mV) was transmitted through the electrodes until a mild twitch of the vertebral body of the animal was observed. One hundred SCEP responses were averaged for each rat to obtain high-quality waveforms for the SCEP signals.

**Histological analysis**

At 8 weeks after SCI, all rats were deeply anesthetized with an adequate dose of 1% Pentobarbital Sodium (40-45mg/kg) and were transcranial perfused with 250 mL of 0.9% normal saline. Animals were perfused with 300 mL of 4% paraformaldehyde (PFA) in 0.1 M phosphate buffer solution (PBS; pH 7.4). The T8-T11 cord segments were dissected based on the dorsal spinal root count, post fixed overnight in 4% PFA, and soaked at 4°C overnight in 10% sucrose followed by 30% sucrose. The specimens were embedded in optimal cutting temperature compound, frozen at -20°C and sliced at a thickness of 20 μm in the longitudinal or transverse plane.

To visualize the cavity area, animals were killed for haematoxylin-eosin (HE) staining. The T8-T11 longitudinal spinal cord sections from each group were stained with HE according to standard protocols and observed under brightfield microscope. For neuron counting, transverse sections of the injured spinal cord were used to stain neurons with Nissl. Sections at 2 mm rostral and caudal to the lesion epicentre were counted for each rat. The numbers of positively stained cells were counted and averaged per section in a blinded manner.

**Magnetic Resonance Imaging (MRI)**

MRI was performed using a 3.0 Tesla Siemens Verio MR scanner equipped with high-performance gradient coils at 8 weeks postinjury. A special eight-channel wrist coil was used to image the rats. Five animals were observed for each group. For scanning, the rats were subjected to general anaesthesia and each animal was subsequently placed in the ventral recumbent position using a surface coil for spinal cord measurements. T1- and T2-weighted magnetic resonance (MR) images of the spinal region were reconstructed in the sagittal plane to visualize the extent of the lesion. The cavity volume of the injured spinal cord was evaluated by contrasting the normal T8-10 spinal cord diameter in the sagittal view (2.2 ±0.3 mm from our measurement) ^9,10^.

**Nissl staining**

Nissl staining was performed on transverse sections rostral to the epicenter with 0.1% Cresyl violet (Sigma-Aldrich, C5042) for 20 min at 37 °C. After rinsing in distilled water, the stained sections were differentiated in 95% ethyl alcohol. Then, the sections were dehydrated in increasing concentrations of ethyl alcohol and cleared in xylene. Subsequently, these sections were imaged and quantified at high magnification. A total of five sections were quantified for each rat, and the average number of each group was calculated to determine neuronal survival.

**Statistical analysis**

Statistical significance was analysed via one-way analysis of variance with Levene’s test for homogeneity of variance, followed by the Bonferroni post hoc test based on the comparison to be made and the statistical indication of each test. P values less than 0.05 were considered significant. Analyses were per-formed using SPSS software for Windows, version 16.0.

**Reference**

1 Chen, N. N. *et al.* Targeted Inhibition of Leucine-Rich Repeat and Immunoglobulin Domain-Containing Protein 1 in Transplanted Neural Stem Cells Promotes Neuronal Differentiation and Functional Recovery in Rats Subjected to Spinal Cord Injury*. *Crit Care Med* **44**, E146-E157, doi:10.1097/Ccm.0000000000001351 (2016).

2 Zhao, X. Y. *et al.* Lentiviral vector delivery of short hairpin RNA to NgR1 promotes nerve regeneration and locomotor recovery in injured rat spinal cord. *Sci Rep-Uk* **8**, doi:Artn 5447

10.1038/S41598-018-23751-2 (2018).

3 Li, X. *et al.* Wnt4-modified NSC transplantation promotes functional recovery after spinal cord injury. *Faseb J* **34**, 82-94, doi:10.1096/fj.201901478RR (2020).

4 Liu, W. T. *et al.* MiRNA-141 and miRNA-200b are closely related to invasive ability and considered as decision-making biomarkers for the extent of PLND during cystectomy. *Bmc Cancer* **15**, doi:ARTN 92

10.1186/s12885-015-1110-7 (2015).

5 Yang, J. *et al.* miR-200b-containing microvesicles attenuate experimental colitis associated intestinal fibrosis by inhibiting epithelial-mesenchymal transition. *J Gastroen Hepatol* **32**, 1966-1974, doi:10.1111/jgh.13797 (2017).

6 Shang, Y. Y. *et al.* HIF-1 alpha/Ascl2/miR-200b regulatory feedback circuit modulated the epithelial-mesenchymal transition (EMT) in colorectal cancer cells. *Exp Cell Res* **360**, 243-256, doi:10.1016/j.yexcr.2017.09.014 (2017).

7 Kanekiyo, K. *et al.* Effects of Multiple Injection of Bone Marrow Mononuclear Cells on Spinal Cord Injury of Rats. *J Neurotraum* **34**, 3003-3011, doi:10.1089/neu.2016.4841 (2017).

8 Wu, H. F. *et al.* The promotion of functional recovery and nerve regeneration after spinal cord injury by lentiviral vectors encoding Lingo-1 shRNA delivered by Pluronic F-127. *Biomaterials* **34**, 1686-1700, doi:10.1016/j.biomaterials.2012.11.013 (2013).

9 Simard, J. M. *et al.* MRI evidence that glibenclamide reduces acute lesion expansion in a rat model of spinal cord injury. *Spinal Cord* **51**, 823-827, doi:10.1038/sc.2013.99 (2013).

10 Ohta, K., Fujimura, Y., Nakamura, M., Watanabe, M. & Yato, Y. Experimental study on MRI evaluation of the course of cervical spinal cord injury. *Spinal Cord* **37**, 580-584, doi:DOI 10.1038/sj.sc.3100881 (1999).

**Supplementary Fig. 1. Wnt5a promotes neural differentiation in NSC**


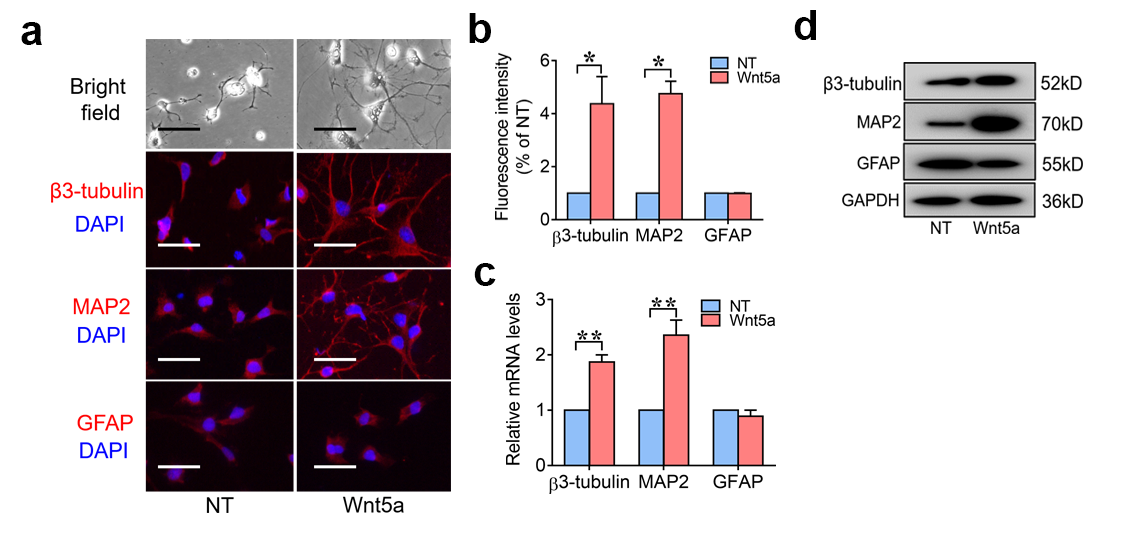


**Fig. S1: Wnt5a promotes neuronal differentiation in NSC.** **(a and b)** Bright fields and Immunofluorescent staining of NSC stimulated with Wnt5a. Bar = 5m **(c and d)** RT-qPCR and Western blot analyses of neurogenesis markers expressions in NSC stimulated with Wnt5a. (The data are presented as the means ± SD from one representative experiment of three independent experiments performed in triplicate. * P < 0.05 compared between groups; ** P < 0.01 compared between groups.)

**Supplementary Fig. 2. miRNA200b-3p promotes neural differentiation in NSC**


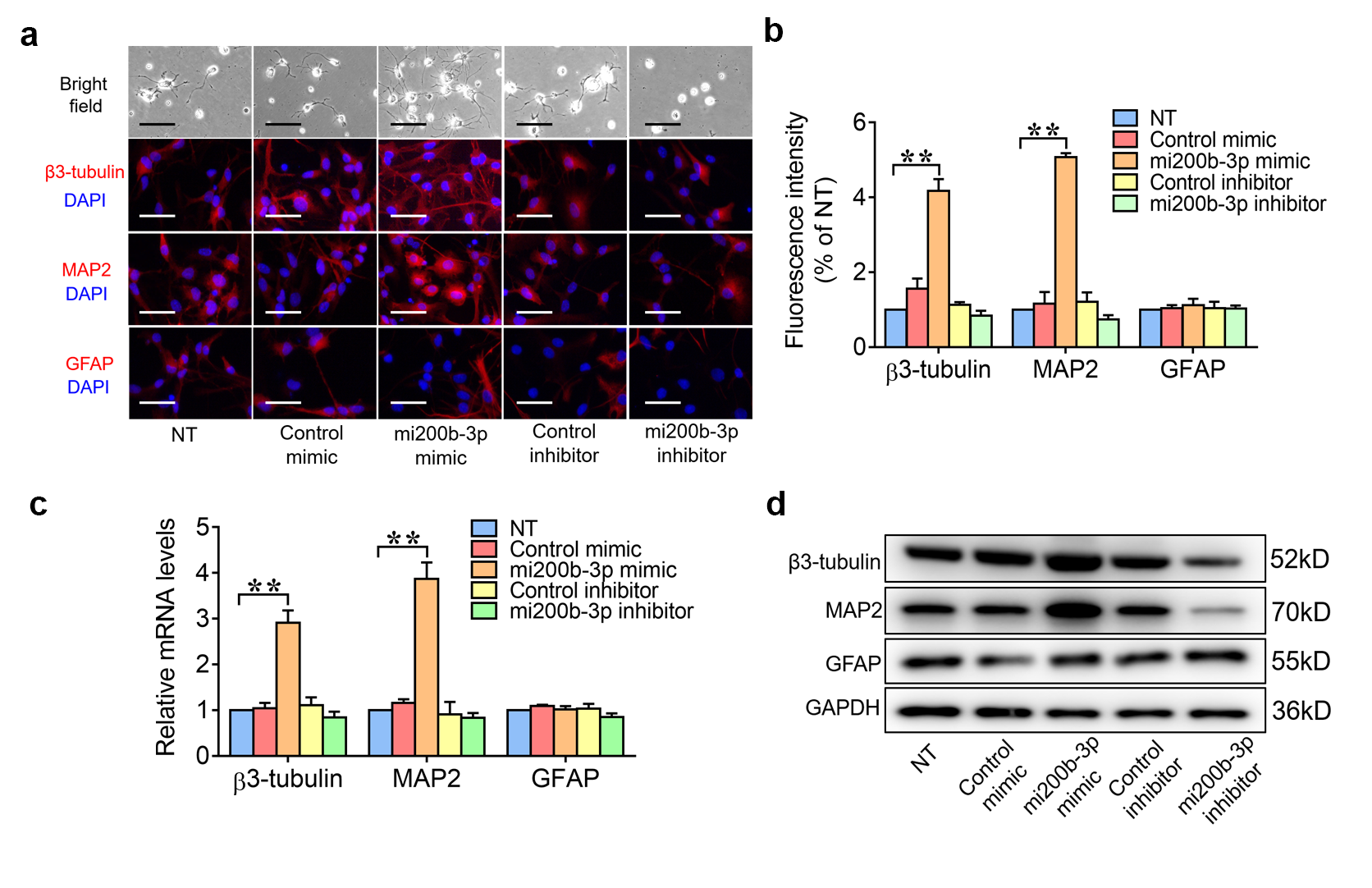


**Fig. S2: mi200b-3p promotes neural differentiation in NSC. (a and b)** Bright fields and Immunofluorescent staining of NSC stimulated with mi200b-3p mimic and inhibitor. **(c and d)** RT-qPCR and Western blot analyses of neurogenesis markers expressions in NSC transfected with mi200b-3p mimic and inhibitor. (The data are presented as the means ± SD from one representative experiment of three independent experiments performed in triplicate. ** P < 0.01 compared between groups.)

**Supplementary Fig. 3. The differentiation status of the transplanted NSC at the injured site after SCI**


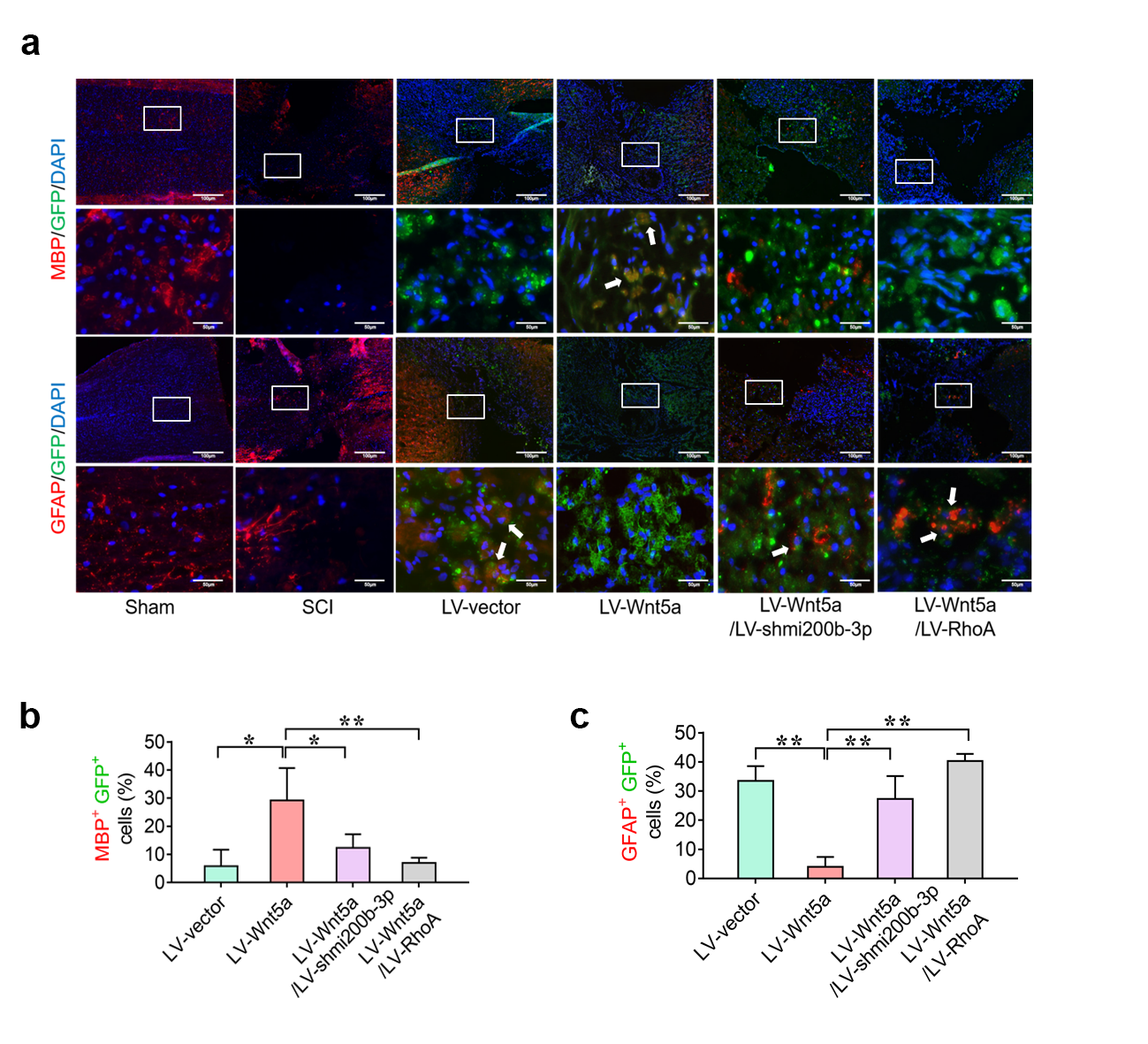


**Fig. S3. The differentiation status of the transplanted NSC at the injured site after SCI. (a)** Immunofluorescent staining of the spinal cord in different groups. The white boxes indicated the images of higher magnification. The white arrow pointed out the co-localizing cells. **(b and c)** Quantification of Immunofluorescent staining. * P < 0.05 compared between groups; ** P < 0.01 compared between groups.

**Supplementary Table 1. Primers for qRT-PCR analysis of gene expression**

**Table S1: Primer sequence for RT-qPCR**

| Gene | Forward 5’-3’ | Reverse 3’-5’ |
| --- | --- | --- |
| β3-tubulin | GCAACTATGTGGGGGACTCGG | CCAGCACCACTCTGACCGAAG |
| MAP2 | GTTGGGCAGTGATTACTACGA | TTCAGGTAACTCGGACGGATG |
| GFAP | GCCCACCAAACTGGCTGAC | CTTGGACCGATACCACTCTTCT |
| Wnt5a | CCACAAGAGACAGCTAGGGC | CACGTTGGAACTGGCTGTTG |
| mi200b-3p | CTCAACTGGTGTCGTGGAGTCG  GCAATTCAGTTGAGGTCATCAT | ACACTCCAGCTGGGTAATACTGC  CTGGTAAT |
| RhoA | CTTCGGAATGATGAGCACACA | CACTCCGTCTTTGGTCTTTGC |
| Rock1 | GCGGTGATGGCTATTATGGA | GCTACAGTGTCTCGGAGCGTTT |
| β-Actin | GCCCATCTATGAGGGTTACGC | TAATGTCACGCACGATTTCCC |
